# A Spiny Climbing Robot with Dual-Rail Mechanism

**DOI:** 10.3390/biomimetics8010014

**Published:** 2023-01-01

**Authors:** Yanwei Liu, Hao Wang, Chongyang Hu, Qiang Zhou, Pengyang Li

**Affiliations:** 1 School of Mechanical and Precision Instrument Engineering, Xi’an University of Technology, Xi’an 710048, China; 2 School of Electrical and Control Engineering, Shaanxi University of Science and Technology, Xi’an 710021, China

**Keywords:** climbing robot, spiny track, rough surfaces, dual-rail mechanism

## Abstract

Easy detachment is as important as reliable an attachment to climbing robots in achieving stable climbing on vertical surfaces. To deal with the difficulty of detachment occurring in wheeled and track-type climbing robots using bio-inspired spines, a novel climbing robot utilizing spiny track and dual-rail mechanism is proposed in this paper. The spiny track consists of dozens of spiny feet, and the movement of each spiny foot is guided by the specially designed dual-rail mechanism to achieve reliable attachment and easy detachment. First, the design of the climbing robot and the dual-rail mechanism are presented. Then, the dual-rail model is constructed to analyze the attaching and detaching movements of the spiny feet, and a mechanical model is established to analyze the force distribution on the spiny track. Finally, a robot prototype is developed, and the analysis results are verified by the experiment results. Experiments on the prototype demonstrated that it could climb on various rough vertical surfaces at a speed of 36 mm/s, including sandpaper, brick surfaces, concrete walls with pebbles, and coarse stucco walls.

## 1. Introduction

Climbing robots could be applied in space exploration, reconnaissance, disaster rescue, and other fields due to their advanced locomotion capacity on steep or even vertical surfaces [1]. To climb and maintain on vertical surfaces without falling off, the reliable attachment is very important for climbing robots. There are several means to generate the adhesion forces needed for climbing, including suction adhesion [2,3,4], magnetic adhesion [5,6,7], electrostatic adhesion [8,9,10,11], gecko-inspired dry adhesive [12,13,14,15,16], and insect-inspired spines [17,18,19,20,21]. Suction adhesion is widely used in climbing robots, but it is not suitable for climbing on rough surfaces due to gas leakage. Magnetic adhesion can provide high adhesion force, but it is suitable only for ferromagnetic surfaces. Electrostatic adhesion is suitable for many materials, but its power consumption is higher. While the gecko-inspired dry adhesive does not need high power to generate adhesion, the current artificial dry adhesives could be easily polluted and then fail when climbing on rough and dusty surfaces. Insect-inspired claws or spines could engage with asperities on rough surfaces to generate adhesion force. It is suitable for the rough and dusty outdoor environment.

Not only reliable attachment but also easy detachment is important in achieving stable climbing performance. In the design of climbing robots, the detachment of robots’ feet should be easy and rapid for efficient locomotion. In nature, because insects possess directional claws and geckos possess directional branched setae arrays on their feet, they can easily and rapidly switch their feet’s adhesion statuses by changing the motion directions of their feet. Inspired by insects’ claws and their attaching and detaching movements, several climbing robots have been developed for vertical rough surfaces. Most of these climbing robots employed legged locomotion, and their feet could easily detach from the climbing surfaces by manipulating the movements of their legs or feet. Asbeck et al. proposed a legged robot with six complaint spiny feet, SpinybotII, which was capable of climbing on hard and rough concrete and brick surfaces [17]. Each spiny foot in SpinybotII was driven by an RC servo to achieve attachment and detachment. Ji et al. developed a quadruped robot utilizing flexible pads and claws, and each foot was driven by one actuator to achieve attaching and detaching movements [18]. Robots Rise V2 [20] and LWbot [21] employed four-bar linkage mechanisms to generate the proper foot motion trajectory for attachment and detachment. Except for these directional spiny feet, some spiny grippers with opposed gripping mechanisms were designed for climbing robots to generate high adhesion force [22,23,24,25,26,27,28]. These spiny grippers consist of two or more spiny feet that move inwards to attach on and outwards to detach from surfaces.

Different from the alternative climbing gait adopted by legged robots, wheeled and track-type climbing robots with minimal actuators are simpler and more effective. Several climbing robots using spiny wheels have been developed for rough vertical surfaces [29,30,31]. However, the rotary motion of the spiny wheel makes it difficult for spines to detach from rough surfaces, and spines might be stuck in cracks. Compared with legged and wheeled climbing robots, track-type climbing robots could achieve more stable attachment due to the larger contact aera or more adhesion units. In addition, several track-type climbing robots using suction cups [3,4] or magnetic adsorption [5] or electrostatic adhesion [8,9] or dry adhesives [12,13] have been developed. However, track-type climbing robots utilizing spines are not common due to the detaching difficulty of spines caused by the rotary motion of spiny track.

In our previous studies, a track-type inverted climbing robot with spiny grippers was developed [32], and the opposed gripping mechanism was employed in the robot to deal with the detaching difficulty. In this paper, a novel dual-rail mechanism is introduced in the design of track-type climbing robots to guide the attaching and detaching movements of the spiny feet.

The reminder of the paper is as follows. Section 2 presents the detailed design of the track-type climbing robot and the dual-rail mechanism. In Section 3, the attaching and detaching movements of the spiny feet while the robot is climbing are analyzed by a dual-rail model, and the force distribution on the spiny track is analyzed. Section 4 gives the experimental results, and Section 5 presents conclusions.

## 2. Robot Design

### 2.1. Overall Design of Robot

**Figure 1:**
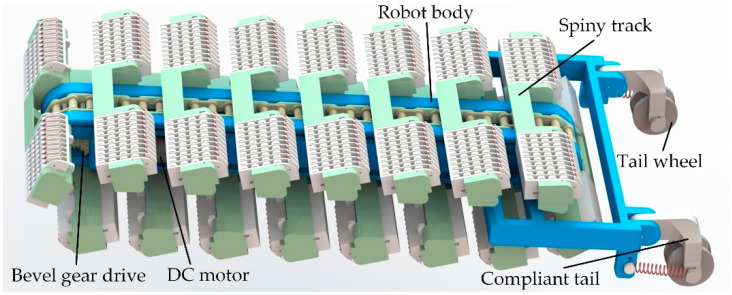
Schematic diagram of the track-type climbing robot.

In this section, a track-type climbing robot using insect-inspired compliant spines and dual-rail mechanism is proposed. As shown in Figure 1, the robot is composed of a main body, a spiny track, and two compliant tails. The compliant tails are designed to provide the preload force for the spiny foot located at the front of the spiny track and reduce the normal adhesion force required on spiny track to balance the pitch-back moment caused by the robot weight. The tail is equipped with a wheel to decrease the friction force. A single DC motor is used to drive the spiny track through a bevel gear transmission and sprocket mechanism.

### 2.2. Design of Spiny Track

**Figure 2:**
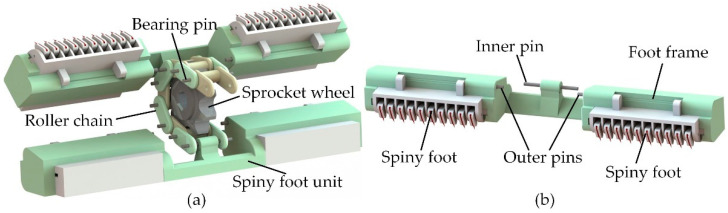
Mechanical configuration of the spiny track. (**a**) Two spiny foot units connected through roller chain; (**b**) structure of the spiny foot unit.

The spiny track is the critical adhesion component for maintaining the robot on vertical rough surfaces. Using the spiny track, the robot can achieve a continuous climbing motion. The spiny track is composed of sixteen spiny foot units. As shown in Figure 2a, these spiny foot units are linked by a roller chain that is driven by a sprocket wheel. As shown in Figure 2b, each spiny foot unit possesses two spiny feet. The spiny foot units and roller chain are connected by inner pins.

**Figure 3:**
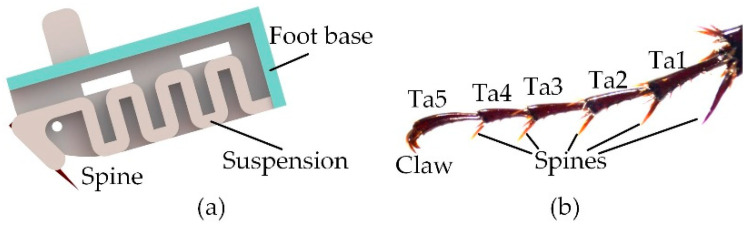
The bio-inspired spiny toe. (**a**) The detailed structure of a compliant spiny toe; (**b**) the flexible tarsal chain of the insect *Serica Orientalis* Motschulsky. (**b**) is reproduced from [32] with permission (Copyright © 2020, Jilin University).

The compliant mechanism of the spiny foot plays an important role in the design of climbing robots with bio-inspired miniature spines. A spiny foot possesses ten bio-inspired spiny toes, and its compliant is achieved through the specially designed spiny toes. The detailed structure of a bio-inspired spiny toe is shown Figure 3a. The spine connects with the foot base through a spring-like compliant suspension. It is inspired by the flexible tarsal chain of the insect *Serica Orientalis* Motschulsky, which has been studied in our previous works [29,32] (Figure 3b). The compliant suspension is important in improving the adhesion performance of a foot with many spines. Its normal flexibility causes more spines to be able to contact the irregular surface, and its elastic deformation in tangential direction makes it possible for more spines to engage on the surface asperities and share the payload.

### 2.3. Dual-Rail Mechanism

**Figure 4:**
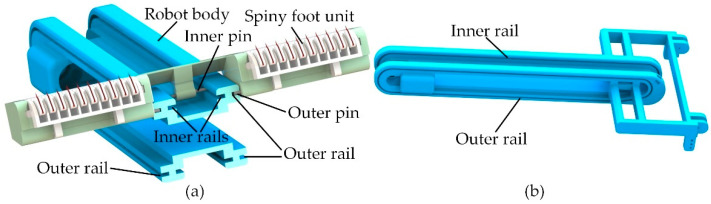
The dual-rail mechanism. (**a**) Mechanical configuration of the dual-rail mechanism; (**b**) slot-shaped endless rails in robot body.

In order to achieve reliable attachment and easy detachment, a dual-rail mechanism was proposed. As shown in Figure 4a, the dual-rail mechanism consists of a spiny foot unit and two endless rails in the robot body. As shown in Figure 4b, the slot-shaped endless rails symmetrically distributed on the outsides of the robot body are called outer rails, and the slot-shaped endless rails symmetrically distributed on the inner sides of the robot body are called inner rails. The bearing pins in the roller chain and the inner pin in the spiny foot unit are constrained by the inner rails to allow the spiny track to move along the endless inner rail. The outer pins in the spiny foot are constrained by the outer rails to change the orientations of the spiny feet during climbing. It means that all spiny foot units move along the outer rails and inner rails, and their movements are guided by the dual-rail mechanism.

## 3. Modeling and Analysis

### 3.1. Attaching and Detaching Movements Analysis

**Figure 5:**
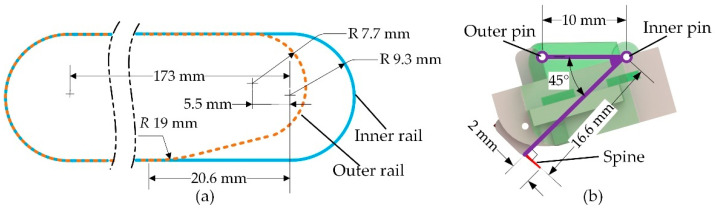
Special designed dual-rail mechanism. (**a**) Detailed design of the outer rail and inner rail; (**b**) detailed design of the spiny foot unit.

**Figure 6:**
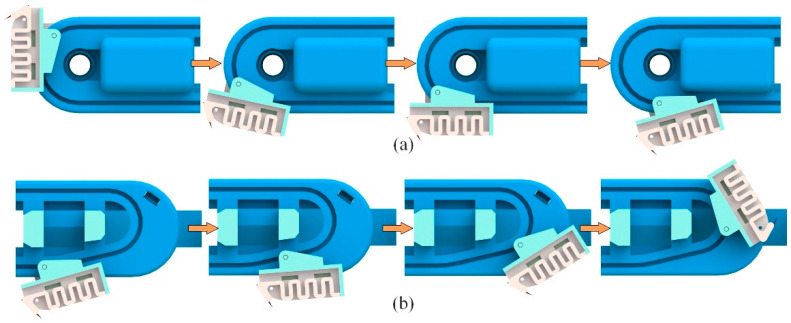
Schematic diagram of attaching and detaching movements of spiny feet. (**a**) Attaching movements; (**b**) detaching movements.

The spiny foot’s movement while the robot is climbing is determined by the shapes of the outer rail and inner rail. Using a special designed dual-rail mechanism, the spiny foot is able to achieve attaching and detaching movements similar to those of insects. Figure 5 shows the detailed design of the dual-rail mechanism. As shown in Figure 5a, the inner rail is a rectangle with two arc ends, and one end of the outer rail is specially designed according to the detaching trajectory of the spine tip. As shown in Figure 5b, the spiny foot unit could be simplified as a linkage with two sliding pins, which move along the outer rails and the inner rail, respectively. With the special designed dual-rail mechanism, the attaching and detaching movements are illustrated in Figure 6. As shown in Figure 6a, the spiny foot rotates anti-clockwise around the inner pin to approach and engage with the target wall surface. As shown in Figure 6b, the spiny foot first rotates clockwise around the inner pin to pull off the spine from the wall surface, and then rotates anti-clockwise around the rails to cause the whole spiny foot to move away from the surface. The first part of the detaching movement is the reverse process of the attaching movement. It means the spiny foot could be easily detached from the wall surface.

**Figure 7:**
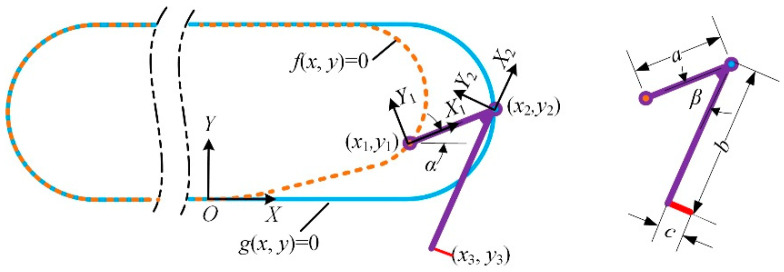
Dual-rail mechanism model.

The easy detachment of the spiny foot is critical in the design of track-type climbing robot. A theoretical model of the dual-rail mechanism is established to analyze the detachment of the spine foot. As shown in Figure 7 the dual-rail mechanism could be simplified as a linkage moves along the two rails. A coordinate system is fixed on the robot’s body, and the coordinate origin is defined as the point where the outer rail starts to leave the inner rail. The curve functions of the outer rail and inner rail are described as *f*(*x*, *y*) and *g*(*x*, *y*), respectively. The length of linkage is constant *a*. One endpoint of the linkage is located on the outer rail, and another endpoint of the linkage is located on the inner rail, and their coordinate values (*x*_1_, *y*_1_) and (*x*_2_, *y*_2_) should satisfy the following equation:

(1) $$\{\begin{array}{c} f(x_{1},y_{1})=0\\ g(x_{2},y_{2})=0\\ {\left(x_{1}-x_{2}\right)}^{2}+{\left(y_{1}-y_{2}\right)}^{2}=a^{2} \end{array}$$

The coordinate values of the spine tip can be calculated with the following equation:

(2) $$\left[\begin{array}{c} x_{3}\\ y_{3} \end{array}\right]=R(\alpha +\beta )\left[\begin{array}{c} -b\\ -c \end{array}\right]+\left[\begin{array}{c} x_{2}\\ y_{2} \end{array}\right]=\left[\begin{array}{cc} \cos(\alpha +\beta ) & -\sin(\alpha +\beta )\\ \sin(\alpha +\beta ) & \cos(\alpha +\beta ) \end{array}\right]\left[\begin{array}{c} -b\\ -c \end{array}\right]+\left[\begin{array}{c} x_{2}\\ y_{2} \end{array}\right]$$

where R (*α* + *β*) is the rotation matrix, *c* is the length of the spine, *b* and *β* are the constant parameters of the spiny foot unit, and the orientation angle of the linkage can be calculated with the following equation:

(3) $$\alpha =\mathrm{atan}2(y_{2}-y_{1},x_{2}-x_{1})$$

**Figure 8:**
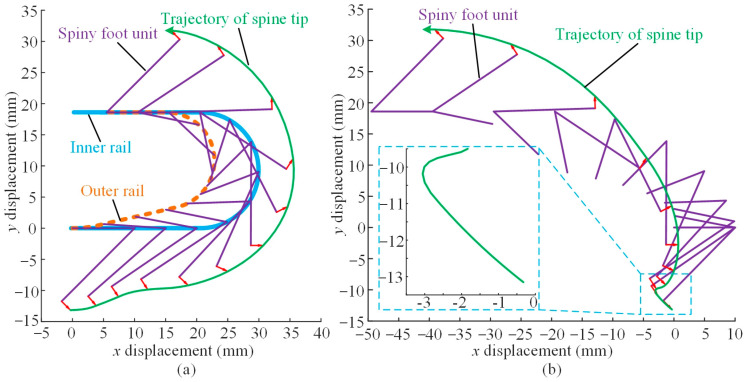
Analysis results of spiny foot’s detachment. (**a**) Detachment with robot body fixed; (**b**) detachment during climbing.

Combining Equations (1)–(3), we can get the detaching movements of the spiny foot and the trajectory of a spine tip during detachment. The analysis is based on the design parameters of the dual-rail mechanism shown in Figure 5, and the analysis results are shown in Figure 8. The green solid lines represent the trajectories of the spine tip during detachment. Figure 8a illustrates the detaching movement with the robot fixed. The analysis results are consistent with the process shown in Figure 6b. First, the spiny foot moves rightward, and it rotates clockwise around the inner pin to lift up the spine tip. Then, the whole spiny foot moves upward. Finally, the spiny foot rotates anti-clockwise around the rails to the upper position and starts to move leftward. In this case, we assume that the robot body is fixed and the spiny foot moves along rails. However, the robot body moves forward when climbing on the wall surfaces. Figure 8b shows the analysis results of the spiny foot’s detachment during climbing. In this case, we assume that the spiny track adheres to the wall surface, the spiny feet in contact with the wall is fixed, and the robot body moves with the motion of the spiny track. The green solid line illustrates the real detaching trajectory of a spine tip while the robot is climbing. During detachment, the spine foot unit rotates clockwise around the inner pin, and the spine tip is lifted upward about 3 mm to detach from the surface. Consider the worst case that the whole spine (2 mm long) has penetrated into the surface; in that event, the spiny foot also has enough detaching distance. Moreover, the direction of the trajectory is basically the same as the orientation of the spine when it is pulled out. In the next detaching action, the spiny foot rotates anti-clockwise, and the spine tip always moves away from the wall. These analysis results demonstrate that the detachment of the spiny foot is easy and reliable.

### 3.2. Quasi-Static Force Analysis

**Figure 9:**
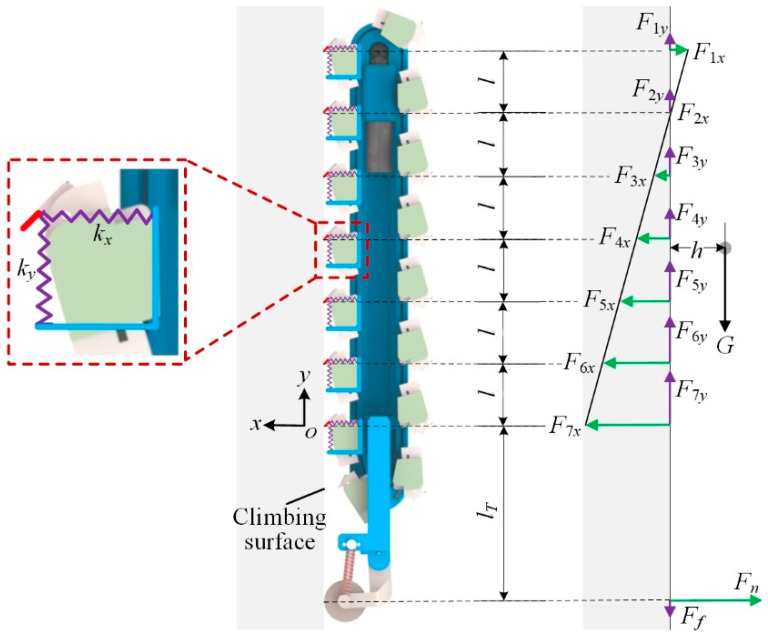
Force analysis of the robot climbing on a vertical surface.

When the robot climbs on vertical surfaces, the spiny feet move with the rotation of the spiny track, and switches between the attached phase and the swing phase. The spiny foot at the front of the robot tries to attach onto the vertical surface, and the spiny foot at the rear of the robot tries to detach from the vertical surface. The total number of attached spiny foot units switch between six and seven during climbing. Figure 9 illustrates the free body diagram of the robot climbing on vertical surfaces with the seven spiny foot units attached. We assume that the robot climbing process is a quasi-static process, and the static balance equation can be obtained:

(4) $$\{\begin{array}{c} \sum_{m=1}^{n}F_{mx}=F_{n}\\ \sum_{m=1}^{n}F_{my}=F_{f}+G\\ \sum_{m=1}^{n}F_{mx}(ml-l+l_{T})=Gh \end{array}$$

where *n* is the number of attached spiny foot units, *F_mx_* and *F_my_* are the normal force and tangential force acted on the *m*-th spiny foot unit, respectively, *F_n_* and *F_f_* are the supporting force and friction force at the tail, *G* is the gravity force, *h* is the distance from the center of gravity to the climbing surface, *l* is the distance between the two adjacent spiny feet, *l_T_* is the distance between the last attached spiny foot and the tail. If the *m*-th spiny foot unit provides adhesion, the normal force *F_mx_* is positive.

Being compliant is a key characteristic of the spiny foot. The spiny foot unit’s tangential stiffness and normal stiffness are *k_y_* and *k_x_*, respectively. The tangential stiffness and normal stiffness are a statistical result, which is related to such factors as wall morphology, the compliant design of a spiny toe, the number of spine toes, the adhesion probability of spine, etc. In the actual robot climbing process, the actual stiffness values vary. We assume that all the attached spiny foot units’ *k_y_* and *k_x_* are equal.

### 3.3. Force Distribution Analysis

**Figure 10:**
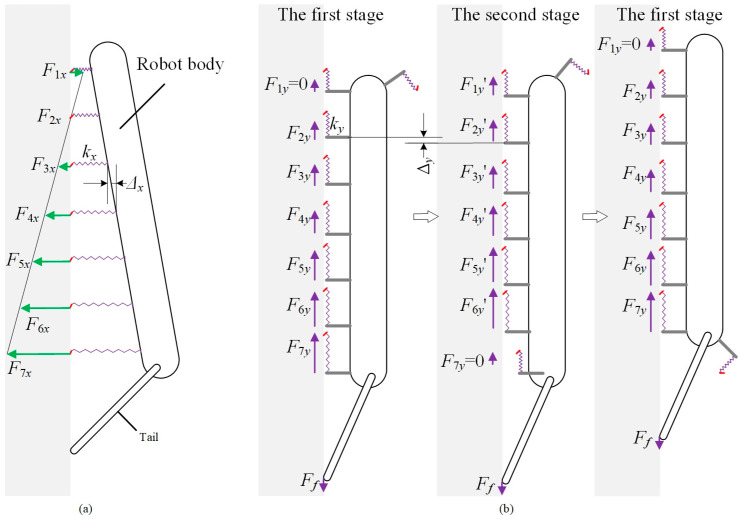
Schematic diagram of force distribution among spiny foot units. (**a**) Normal force distribution; (**b**) tangential force distribution.

For reliable adhesion, the adhesion needed to balance the pitch-back moment and the gravity of the robot should be evenly distributed among the attached spiny feet. The normal force distribution and tangential force distribution are analyzed in Figure 10. As shown in Figure 10a, the first spiny foot unit should be pressed on the climbing surface, and the others provide the adhesion forces. The normal deformations of these attached spiny foot units are equal-difference sequences, and the common difference is Δ*_x_*. It means that the normal forces *F*_1*x*_ to *F_nx_* are equal-difference sequences:

(5) $$F_{(m+1)y}-F_{my}=k_{x}\Delta x$$

where *m* is from 1 to 6. The normal forces could be distributed more evenly by adjusting the supporting force *F_n_* on the tail. According to Equation (4), if the supporting force is too small, it is not enough to overcome the pitch-back moment generated by the robot’s weight. On the contrary, if the supporting force is great, the adhesion needed is greater, and more spiny feet will be under the preload state rather than the adhesive state. It means greater adhesion force should be generated by less spiny feet. Therefore, the supporting force on the tail should be moderate for reliable climbing performance.

The climbing process of the robot is illustrated in Figure 10b for analyzing the tangential force distribution. The climbing process can be divided into two stages. In the first stage, there are seven spiny foot units attached on the climbing surface. The first spiny foot unit just touches the climbing surface, and there is no tangential relative displacement between the spines and the climbing surfaces. As the robot continues to climbing up, the robot switches to the second stage. The last spiny foot unit has detached from the climbing surfaces, and the tangential force *F*_7*y*_ is distributed among the other spiny foot units. All these six spiny foot units have the same deformation increment Δ*_y_*, which means that the tangential force *F*_7*y*_ is evenly distributed among these six spiny foot units. And the same force increment is *F*_7*y*_/6. Then another spiny foot unit touches the climbing surface with the climbing motion of robot, and the robot switches into the first stage again. According to the above analysis, we have the following equation:

(6) $$\{\begin{array}{c} F_{1y}=0\\ F_{(m+1)y}={F_{my}}^{\prime }=F_{my}+\frac{F_{7y}}{6} \end{array}$$

where *m* is from 1 to 6. From Equations (4) and (6), the tangential forces on the attached spiny foot units could be calculated as:

(7) $$F_{my}=\frac{\left(m-1\right)}{6}F_{7y}=\frac{\left(m-1\right)}{21}(G+F_{f})$$

## 4. Results and Discussion

**Figure 11:**
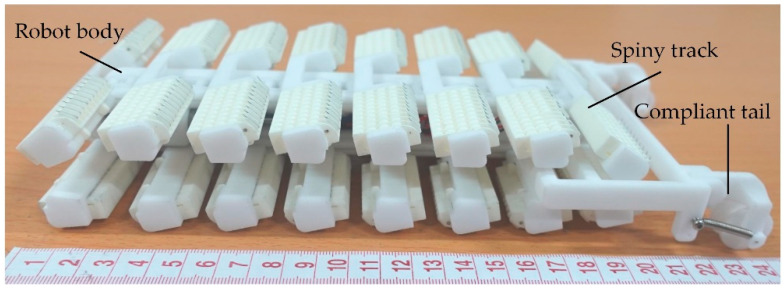
The prototype of the robot.

As shown in Figure 11, a prototype of the track-type robot was fabricated based on the dual-rail mechanism. The majority of the robot was made of nylon material PA2200, using SLS (Selective Laser Sintering) technology. The spines were made from stainless steel disposable acupuncture needles with 300 μm shaft diameters and approximately 10 μm tip radius. The robot prototype is 250 mm (length) × 121 mm (width) × 44 mm (height) in size and 273 g in weight (including an onboard lithium battery). The robot is controlled via a remote radio controller. The performance of the robot prototype was studied through force tests and climbing experiments.

### 4.1. Force Distribution

**Figure 12:**
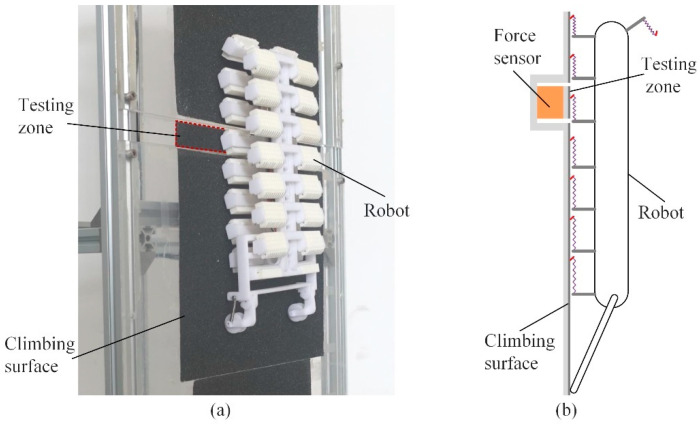
The force-testing platform. (**a**) Prototype of the proposed experimental setup; (**b**) schematic design of the proposed experimental setup.

In order to obtain the contact force between the spiny foot unit and climbing surface during climbing, a force-testing platform was built, as shown in Figure 12. The acrylic plates covered with sandpapers are used as climbing surface, and the climbing surface is divided into three parts. The narrow piece in the middle part, called the testing zone, is fixed on a two-dimension force sensor. While the robot is climbing, the force sensor records the measurements of the tangential force and normal forces acting on the spiny foot unit and the tail.

**Figure 13:**
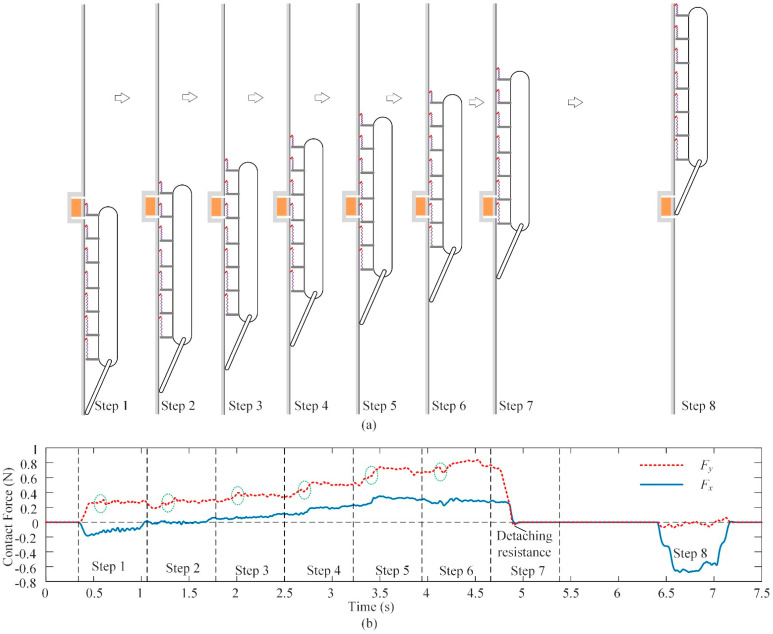
Force-testing results during robot climbing upward. (**a**) Schematic diagram of the force-testing process; (**b**) force-testing results.

As shown in Figure 13a, the testing process mainly consists of eight steps. In the first step, the spiny foot unit gripped onto the test zone. In step 2, a new spiny foot unit started to touch the climbing surface, and the spiny foot unit touching the test zone switched to be the second place. This sequence was repeated, until the spiny foot touching the test zone became the last one and finally detached from the test zone. Both the normal force and the tangential force on the spiny foot unit were measured and recorded during the climbing process. In step 8, the tail moved to the test zone, and the contact forces on the tail were measured and recorded.

Figure 13b illustrates an example of the force-testing results on the 36-grit sandpaper surface. The blue solid line represents the normal force, and the red dashed line represents the tangential force. The result curves could be divided into eight parts, corresponding to the eight climbing steps in Figure 13a. In the first step, the spiny foot is lightly pressed to grip onto the climbing surface. The normal force is small and negative with a maximum value of 0.2 N, and the tangential force increases quickly and then stabilized at 0.28 N. In the second step, the spiny foot moves away from the climbing surface and the preload force disappears, and the tangential force remains stable. From step 3 to step 6, the contact forces increase gradually: the maximum adhesion force is about 0.8 N, while the maximum tangential force is about 0.36 N. In step 7, the spiny foot tries to detach from the climbing surface, and the adhesion force and tangential supporting force finally drop to zero. The normal detaching resistance and tangential resistance are only 0.01 N and 0.02 N, respectively. It means that it is easy for the spiny foot to detach from the climbing surface. In step 8, the wheel on the compliant tail touches the testing zone. The supporting force *F_n_* is about 0.67 N, and the friction force *F_f_* is small enough to be ignored. In general, the normal force and tangential force on the spiny foot unit basically show a linear increasing trend. In addition, there is a sudden change of contact force in each step, as circled by the green dashed lines in Figure 13b. At the moment when the sudden change of contact force appears, the last spiny foot unit detaches from the climbing surface, the robot switches from the first stage to the second stage (as shown in Figure 10b), and the contact forces on the left spiny foot units increase. Moreover, the contact force results also reflect the contact force distribution on the spiny track. The test results of the normal force and tangential force in step 1 to step 7 correspond to the contact forces *F_mx_* and *F_my_* on the seven spiny foot units attached on the climbing surface. The test results of the force distribution are consistent with the analysis results in Section 3.3.

### 4.2. Climbing Performance

**Figure 14:**
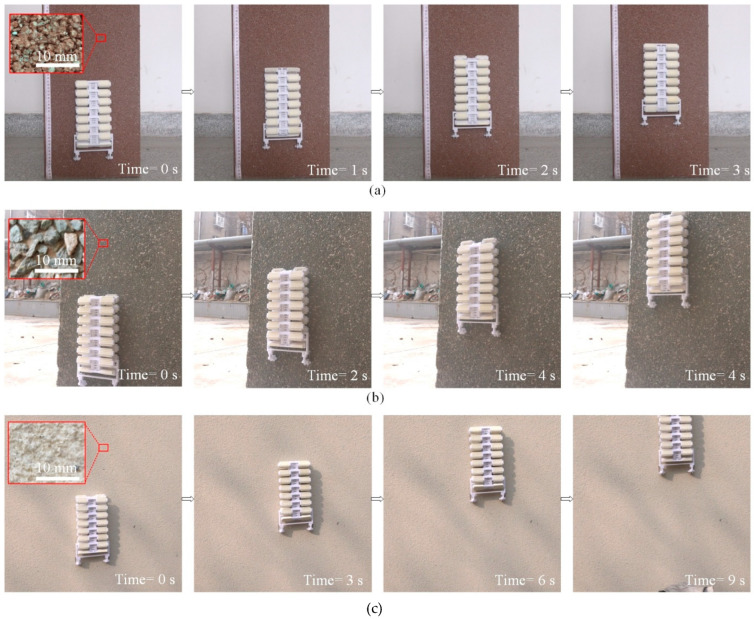
Climbing experiments of the robot prototype on various vertical surfaces. (**a**) Climbing on a brick surface; (**b**) climbing on a concrete wall with pebbles; (**c**) climbing on a coarse stucco wall.

A robot prototype was made to perform on various rough surfaces to evaluate its capacity. All the climbing surfaces are vertical, and the slope angles are 90 degrees. Considering that the climbing performance of the robot is related to some random characteristics of the climbing surfaces, such as the size of particles, the embedded depth, and the distribution of the particles, it is difficult to describe the climbing performance directly by the surface roughness or other parameters. Herein, sandpapers with different meshes are chosen as standards to judge the climbing performance of the robot, and experiment results show that the robot can climb on surfaces rougher than P120 sandpaper surfaces. The average size of the sand particles is about 120 μm. Climbing experiments on the three typical rough surfaces are illustrated in Figure 14, as well as the surface morphology. The average sizes of the particles on the brick surface, the concrete wall with pebbles, and the coarse stucco wall are about 2 mm, 3 mm, and 1 mm, respectively. As shown in Figure 14a, the climbing speed of the robot on a vertical brick surface is about 36 mm/s. The load capacity of the robot on the brick surface is 200 g, and the climbing speed is 20 mm/s with load. Benefiting from track-type design and dual-rail mechanism, the robot can climb on actual vertical exterior walls. Experimental results in Figure 14b,c demonstrate its capacity of vertical climbing on a concrete wall with pebbles and a coarse stucco wall, respectively.

### 4.3. Attachment and Detachment of Spiny Feet

**Figure 15:**
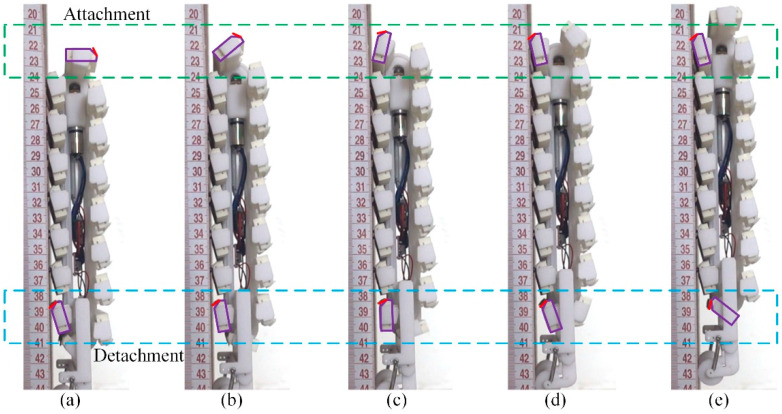
Detachment and attachment of spiny feet during robot climbing. (**a**) The rear spiny foot starts to detach from the climbing surface; (**b**) the rear spiny foot is detaching from the climbing surface; (**c**) the rear spiny foot has detached from the climbing surfaces; (**d**) the front spiny foot starts to touch the climbing surface; (**e**) the front spiny foot has attached on the climbing surface.

The performance of both the attachment and detachment of the spiny feet during the vertical climbing is illustrated in Figure 15. The attaching movements of the front spiny foot are framed with a green dashed rectangle. From Figure 15a–c, the front spiny foot rotates anti-clockwise to approach the climbing surface, and the spiny foot starts to touch the climbing surface in Figure 15d. The attaching movements of the spiny foot are consistent with the analysis results in Figure 6a. The detaching movements of the rear spiny foot are framed with a blue dashed rectangle. From Figure 15a–c, the rear spiny foot rotates clockwise to lift from the climbing surface. These detaching movements are the reverse movements of the attaching process. The whole body of a spine is pulled out from the climbing surface. From Figure 15c–e, the rear spiny foot rotates anti-clockwise to move away from the climbing surface. The experimental results of detachment are consistent with the analysis results in Figure 6a and Figure 8b. The experimental results prove the effectiveness of the dual-rail mechanism proposed in this paper for solving the problem of the detachment of spiny foot in track-type climbing robots. Moreover, the climbing process of the robot is consistent with the analysis results in Figure 10b. There are seven spiny foot units attached on the climbing surface in Figure 15a, and the robot is in the first climbing stage. When the rear spiny foot unit detaches from the climbing surface in Figure 15c, the climbing state of the robot switches to the second. In Figure 15d, when a new spiny foot unit attaches on the climbing surface, the robot switches to the first climbing stage again.

## 5. Conclusions

In this paper, a track-type climbing robot with bio-inspired spiny feet was developed for stable climbing on rough wall surfaces. To deal with the difficulty of detachment occurring in track-type climbing robots, a novel dual-rail mechanism was proposed in the robot design. The mechanical model of the specially designed dual-rail mechanism was established to analyze the attaching movements and detaching movements of a spiny foot. The analysis results and experimental results demonstrated that the spiny feet detached from the climbing surface with the reverse motion of attachment, and the detaching resistance was negligible, which means that the easy detachment of the spiny feet was achieved. Additionally, to study the force distribution among the attached spiny feet, a mechanical model was established. The analysis results of the force distribution showed that the normal force and tangential force were distributed among these spiny feet in the form of equal-difference sequences, and the contact force test results verified the analysis results. Finally, the climbing capacity of the robot prototype was experimentally demonstrated. The robot can achieve stable climbing on diverse rough surfaces, including sandpaper surfaces, brick surfaces, and some real exterior walls. Its climbing speed is about 36 mm/s, and its payload capacity can reach 200 g.

The climbing performance of the track-type robot on some real exterior walls has been demonstrated in this paper. However, the locomotion function of the robot is still limited for practical applications. In the future works, the bio-inspired spiny foot will be optimized for stronger adhesion, and a robot with multi-linked spiny tracks will be developed to improve its locomotion ability, including ground-to-wall transitions, turning function, and lateral climbing ability.

## Data Availability

Not applicable.
